# Editorial: Synaptic Plasticity and the Neurobiology of Learning and Memory

**DOI:** 10.3390/cells15131169

**Published:** 2026-06-26

**Authors:** Anna Panuccio, Laura Petrosini, Erica Berretta

**Affiliations:** 1 IRCCS Fondazione Santa Lucia, 00179 Rome, Italy; anna.panuccio@gmail.com (A.P.); l.petrosini@hsantalucia.it (L.P.); 2 Department of Human Sciences, Università degli Studi Guglielmo Marconi, 00193 Rome, Italy

## 1. Introduction

Synaptic plasticity, the activity-dependent modification of synaptic strength, is widely recognized as the principal cellular substrate of learning and memory. Long-term potentiation (LTP) and long-term depression (LTD), first described decades ago, remain paradigmatic examples of how patterns of neural activity can be durably encoded at the synapse through changes in receptor trafficking, spine morphology and number, and gene expression. Yet the molecular and cellular landscape of synaptic plasticity has expanded far beyond the classical “neuron-centric” framework. It is now evident that astrocytes, microglia, and immune mediators are active participants in regulating synaptic function, and that disruption of synaptic homeostasis is not limited to narrowly defined cognitive disorders but also extends to motivation, emotional regulation, and neurodevelopment.

The Special Issue of Cells, “Synaptic Plasticity and the Neurobiology of Learning and Memory”, brings together three original research articles [1,2,3] and two comprehensive reviews [4,5] that address several frontier areas of the field: the involvement of reward circuits and endocannabinoid signaling in synaptic dysfunction associated with Alzheimer’s disease (AD); the transcriptional consequences of inherited metabolic disease on prefrontal cortical networks; the post-transcriptional regulation of synaptic identity molecules; and the emerging roles of astrocytes and neuroinflammatory mediators as modulators of plasticity. In what follows, we discuss each contribution in turn, highlighting the methodological approaches, key findings, and implications for understanding synaptic plasticity in health and disease.

## 2. Motivational Deficits and Synaptic Dysfunction in Alzheimer’s Disease: A Role for Palmitoylethanolamide

Alzheimer’s disease is a progressive neurodegenerative disorder whose clinical manifestations span multiple cognitive domains, including memory, executive functions, language, and visuospatial abilities, reflecting the widespread and evolving nature of the underlying neuropathological changes. Yet mounting evidence points to a far broader neurobiological picture in which affective, motivational, and reward-related disturbances (including apathy, anhedonia, and impaired reward processing) play a central and often early role, preceding overt cognitive decline and reflecting dysfunction of mesolimbic circuits well beyond the classical cortico-hippocampal axis. The neuropsychiatric dimensions of AD are not merely epiphenomena; they profoundly affect quality of life, caregiver burden, and, importantly, the capacity of patients to engage in cognitively stimulating activities that might otherwise support cognitive reserve.

Panuccio and colleagues [1] addressed this underexplored dimension of AD using the Tg2576 transgenic mouse model, which overexpresses a mutant form of the human amyloid precursor protein (APP) and develops progressive amyloid pathology reminiscent of the human disease. Their study focuses specifically on incentive motivation, i.e., the drive to expend effort in pursuit of reward, and its neurobiological substrates, including synaptic plasticity mechanisms in reward-related circuits. A particular focus was placed on the entorhinal cortex (EC), a bidirectional relay among the hippocampus, amygdala, and prefrontal cortex (PFC) that encodes not only spatial representations but also the motivational salience of contextual stimuli, thereby serving as a pivotal node at the intersection of memory, affect, and motivated behavior. Incentive motivation was assessed using the conditioned place preference (CPP) paradigm, a Pavlovian contextual-association learning task that engages the hippocampus, PFC, and EC. The authors demonstrate that Tg2576 mice exhibit significant motivational deficits alongside impaired synaptic plasticity, and that these alterations can be rescued by sustained infusion of palmitoylethanolamide (PEA), an endogenous lipid belonging to the N-acylethanolamide family with well-known anti-inflammatory, neuroprotective, immunomodulatory, and neurotrophic properties. In particular, Tg2576 placebo-treated mice failed to acquire a preference for the chocolate-paired chamber despite residual food consumption during conditioning, indicating a loss of motivational value assigned to the reward rather than a generalized sensory or motor impairment. Chronic PEA administration via sustained-release subcutaneous pellets over six months fully reinstated CPP performance in Tg2576 mice without affecting wild-type animals, a genotype-specific rescue that speaks directly to PEA relevance in disease.

PEA exerts its effects primarily through peroxisome proliferator-activated receptor alpha (PPARα) and, to a lesser extent, through indirect modulation of the endocannabinoid system via an “entourage effect”. Its ability to reduce neuroinflammation, limit microglial activation, and restore lipid homeostasis makes it a particularly attractive candidate for intervention in a disease context where neuroinflammation is increasingly recognized as a pathogenic driver rather than a mere correlate. At the molecular level, PEA restored PPARα expression in the PFC and produced a marked upregulation of brain-derived neurotrophic factor (BDNF), notably even in the absence of a significant baseline BDNF deficit, suggesting a neurotrophic potentiation rather than a mere compensatory rescue. Morphologically, Tg2576 placebo-treated mice exhibited significant dendritic spine loss in the dentate gyrus of hippocampus and in both basal and apical compartments of EC pyramidal neurons. Interestingly, PEA rescued spine density in these regions, with a generalized enhancement of basal dendritic complexity consistent with strengthening of the EC feedforward microcircuits that link contextual representations with reward retrieval.

The findings of Panuccio et al. [1] are thus significant on multiple levels: they reinforce the importance of motivational neuroscience in the study of AD, implicate the PEA–PPARα–BDNF axis as a mechanistic substrate for the concurrent rescue of behavioral and synaptic phenotypes, and provide preclinical support for lipid-based neuromodulation as a therapeutic strategy targeting the non-cognitive symptoms of neurodegeneration. These results invite further investigation into the circuit-level mechanisms by which PEA restores incentive motivation, and into the translational potential of PEA-based interventions in early-stage AD.

## 3. Metabolic and Genetic Determinants of Synaptic Gene Expression: Prefrontal Transcriptomics in Phenylketonuria

A fundamental challenge in preclinical neuroscience is the extent to which findings in animal models generalize, both across genetic backgrounds within a species and ultimately to the human condition. This issue is particularly acute in the study of inherited metabolic disorders, where the genetic background of the model organism can substantially modify the phenotypic expression of a given mutation, raising questions about the translational relevance of preclinical findings.

Fiori and colleagues [2] tackled this challenge directly in the context of phenylketonuria (PKU), an autosomal recessive disorder caused by loss-of-function mutations in the phenylalanine hydroxylase (PAH) gene, leading to hyperphenylalaninemia (HPA) and its neurotoxic consequences. Despite decades of neonatal screening and dietary intervention, early-treated PKU patients continue to show mild but consistent impairments in executive functions, working memory, and inhibitory control, a neuropsychological profile that points specifically to PFC dysfunction. Two widely used mouse strains carrying the same ENU-induced PAH mutation, BTBR^enu2^ and C57^enu2^, display comparable levels of severe HPA yet diverge markedly in their behavioral phenotypes, with BTBR^enu2^ mice showing a far more severe cognitive impairment than C57^enu2^ mice. Whether this divergence reflects qualitatively different molecular responses to HPA or rather quantitative differences in the same underlying pathological processes remains an open question.

To address this, Fiori et al. [2] performed genome-wide mRNA sequencing of PFC tissue from adult mice of both backgrounds. Differential expression analysis identified 391 differentially expressed genes in the C57 background and 306 in the BTBR background. Critically, 62 genes were commonly modulated across both strains, displaying strong directional consistency: the vast majority were regulated in the same direction regardless of background. Only three genes (Nup62, Psph, and Arhgef2) showed opposite regulation patterns between strains, and the authors noted that each of these encodes a protein with potential relevance to background-specific phenotypic differences: Nup62 is a core component of the nuclear pore complex involved in mRNA export; Psph encodes phosphoserine phosphatase, a key enzyme in L-serine biosynthesis with established links to neurodevelopmental disorders; and Arhgef2 is a Rho guanine nucleotide exchange factor implicated in cytoskeletal dynamics and neuronal morphogenesis.

Among the shared transcriptional changes, the most prominent were the upregulation of aminoacyl-tRNA synthetases and eukaryotic translation initiation and elongation factors across both backgrounds, pointing to a conserved adaptive response to metabolic stress, likely reflecting compensatory transcriptional activation of protein synthesis machinery under conditions of chronically disrupted amino acid balance. Equally notable was the convergent downregulation of myelination-related gene programs in both strains, consistent with the white matter abnormalities repeatedly documented in PKU patients and with prior evidence of altered myelin basic protein expression in BTBR^enu2^ mice. Together, these findings suggest that HPA triggers a shared molecular response in the PFC regardless of genetic background, centered on translational stress and impaired oligodendrocyte function.

The functional significance of this shared transcriptional signature was probed behaviorally in C57^enu2^ mice using the Identical Object Task, a cognitively demanding working memory paradigm that tests PFC-dependent performance by progressively increasing attentional load. While C57^enu2^ mice had previously been shown to perform normally on standard two-object recognition tasks, they failed to discriminate a novel object when four or six identical objects were presented simultaneously, a deficit not observed in wild-type C57 controls, who successfully identified the novel object even with six objects. This previously unreported working memory impairment under high information load extends to the C57 background a cognitive phenotype previously considered exclusive to BTBR^enu2^ mice, and provides behavioral validation of the shared PFC transcriptional dysregulation identified in the sequencing analysis.

Taken together, the findings of Fiori et al. [2] support the view that the phenotypic difference between the two PKU mouse strains is quantitative rather than qualitative: the C57 background confers greater resilience and adaptive capacity in response to HPA, but does not prevent PFC molecular dysregulation or its cognitive consequences when sufficiently challenged. On translational grounds, this convergence strengthens the preclinical relevance of both models and suggests that the spectrum of phenotypes they represent may better capture the wide clinical variability observed in early-treated PKU patients, including those individuals who, despite adequate metabolic control, continue to show subtle but persistent executive deficits in adulthood. These findings open the door to identifying conserved therapeutic targets and may inform the design of interventions to normalize PFC function in PKU patients beyond dietary phenylalanine restriction.

## 4. CB1 Receptor Signaling and the Alternative Splicing of Neurexins: A New Layer of Synaptic Regulation

Neurexins are a family of presynaptic cell-adhesion molecules encoded in mammals by three genes (Nrxn1, Nrxn2, Nrxn3), each of which can be transcribed as either a long (α) or short (β) isoform and is subject to extensive alternative splicing at up to six canonical splice sites (SS1–SS6). This combinatorial diversity generates hundreds of neurexin variants with distinct binding properties for postsynaptic partners, including neuroligins, LRRTMs, dystroglycan, and cerebellins, and is thought to constitute a molecular code that specifies synaptic identity and connectivity. Among the splice sites, SS4 is the most conserved and best characterized: inclusion or exclusion of a 90 bp exon cassette at this site determines the interaction of neurexins with specific postsynaptic ligands, shapes glutamate receptor composition at excitatory synapses, and is dynamically regulated by neuronal activity. Perturbations of neurexin splicing have been linked to autism spectrum disorder, schizophrenia, and other neurodevelopmental conditions, underscoring the functional importance of precise splice-site regulation.

Innocenzi et al. [3] provided a significant new insight into the upstream regulation of neurexin splicing by demonstrating that cannabinoid receptor 1 (CB1) signaling dynamically modulates alternative splicing decisions at SS4 in the hippocampus. Using an ex vivo hippocampal slice preparation, the authors first established that pharmacological modulation of CB1 bidirectionally controls glutamatergic transmission: application of the CB1 agonist ACEA reduced both evoked and spontaneous excitatory postsynaptic currents, while the antagonist AM251 produced the opposite effect, increasing glutamatergic activity. These changes in neural activity were tightly coupled to shifts in the SS4 splicing pattern of Nrxn1–3: AM251 treatment promoted exon inclusion, increasing the proportion of SS4+ variants across all three neurexins, while ACEA promoted exon skipping, shifting the balance toward SS4− variants predominantly in Nrxn1 and Nrxn2. Notably, SS4+ expression was associated with increased neurotransmission and SS4− with reduced neurotransmission, suggesting that CB1-dependent splicing shifts participate in a feedback loop that fine-tunes synaptic strength.

To establish the mechanistic basis of this regulation, the authors turned to mice lacking Slm2, a member of the STAR family of RNA-binding proteins and the principal regulator of SS4 splicing in the adult hippocampus. In Slm2 knockout mice, SS4 alternative splicing was virtually abolished, with constitutive inclusion of the SS4+ exon across all three Nrxn transcripts. Strikingly, the pharmacological modulation of neurotransmission by both ACEA and AM251 was completely suppressed in the absence of SLM2, demonstrating that Nrxn1–3 alternative splicing at SS4 is not merely a correlate of CB1-dependent activity changes but is causally required for the proper response of the hippocampal CA1-subiculum circuit to endocannabinoid signaling.

The authors propose a homeostatic model in which CB1-induced suppression of neurotransmission drives the expression of SS4− variants, which in turn reduce postsynaptic endocannabinoid synthesis and thereby restore excitatory tone; conversely, CB1 inhibition promotes SS4+ inclusion, which may act as a brake to counteract excessive excitatory drive. This bidirectional, activity-dependent splicing program thus constitutes a novel molecular interface linking endocannabinoid signaling to the post-transcriptional regulation of synaptic identity molecules. While CB1 receptors have long been recognized as key regulators of retrograde synaptic signaling, their involvement in shaping the neurexin splice landscape represents a previously unappreciated dimension of endocannabinoid neurobiology, with broad implications for our understanding of how the synaptic chemical milieu influences its structural and functional properties. Future work will need to determine which intracellular signaling cascades downstream of CB1 converge on the SLM2-dependent splicing machinery, and whether this mechanism operates similarly under physiological learning conditions and in pathological states in which endocannabinoid signaling is disrupted.

## 5. Glial Cells, Immune Mediators, and Astrocyte-Mediated Plasticity: Beyond the Neuron

Two comprehensive reviews published in this Special Issue expand the conceptual framework of synaptic plasticity to encompass the critical contributions of non-neuronal cells and immune mediators.

Imbriani et al. [4] provided an authoritative synthesis of the bidirectional communication between the central nervous system and the immune system, with particular attention to how dysregulation of immune mediators disrupts synaptic plasticity in a range of central nervous system disorders. The review opens with a detailed characterization of the main cellular players of the neuroimmune response (microglia, astrocytes, and peripheral immune cells) and their physiological roles in supporting synaptic homeostasis. Under normal conditions, microglia continuously survey the brain microenvironment, promote synaptogenesis and circuit refinement by secreting trophic factors, such as BDNF and IGF-1, and participate in activity-dependent synaptic pruning via complement-mediated mechanisms. Astrocytes, through their role in the tripartite synapse, regulate glutamate reuptake, release gliotransmitters such as D-serine and ATP, and modulate potassium buffering and calcium signaling, all processes essential for synaptic transmission and plasticity. Cytokines, at low and tightly regulated concentrations, participate constructively in synaptic scaling, LTP induction, and neurogenesis; IL-1β supports memory consolidation through astrocyte-mediated BDNF production, while TNF-α regulates homeostatic synaptic scaling and the surface expression of AMPA receptors. The review by Imbriani et al. [4] then examines how this finely tuned neuroimmune balance becomes disrupted in neurodegenerative and neurodevelopmental conditions. In AD, chronic microglial activation driven by Aβ oligomers and tau pathology triggers sustained release of pro-inflammatory cytokines, particularly IL-1β, IL-6, and TNF-α, that impair NMDAR-dependent LTP, facilitate LTD, and promote complement-mediated pathological synapse loss via C1q and C3 tagging. Soluble Aβ oligomers further disrupt glutamatergic signaling by over-activating extrasynaptic NMDARs and mGluR5, driving spine retraction and AMPAR internalization in a self-reinforcing cycle of neuroinflammation and synaptic vulnerability. In Parkinson’s disease, α-synuclein aggregates act as DAMPs that activate microglia via TLR2, sustaining a chronic neuroinflammatory state that impairs synaptic vesicle dynamics, disrupts dopaminergic transmission, and contributes to the early synaptopathy now recognized as a cardinal feature of the disease. The review highlights how, in autism, dysregulated glial signaling during critical developmental windows disrupts synaptic pruning and circuit maturation, as exemplified by defective CX3CR1-mediated microglial pruning, altered TREM2 expression, and excitatory/inhibitory imbalance, in a time- and genotype-dependent manner that shapes phenotypic heterogeneity across the autism spectrum. A central and recurring theme is that neuroinflammation does not typically initiate disease but rather acts as a permissive and amplifying factor that accelerates synaptic dysfunction when chronic or unresolved. Consistent with this view, the authors discuss non-pharmacological multimodal stimulation strategies, including environmental enrichment, physical exercise, and combined cognitive-motor training, as promising complementary approaches to harness neuroimmune modulation and preserve synaptic plasticity across neurological conditions. By framing neuroinflammation as a convergence point across seemingly disparate disorders, Imbriani et al. provide a unifying perspective with significant implications for the development of temporally targeted therapeutic strategies at the neuroimmune interface [4].

Yamamoto and Takano [5] offered a complementary, astrocyte-focused perspective that bridges molecular, cellular, and systems-level analyses of synaptic plasticity. Their review synthesizes the rapidly growing literature on perisynaptic astrocytic processes (PAPs), the highly motile, morphologically specialized extensions of astrocytes that ensheathe synapses and dynamically regulate the synaptic microenvironment. The authors describe how PAPs form structured tripartite synaptic assemblies with presynaptic terminals and postsynaptic densities, controlling glutamate clearance, potassium buffering, and the local availability of gliotransmitters such as D-serine, a co-agonist at NMDA receptors and a critical regulator of LTP induction. Astrocytic calcium signaling, mediated by IP_3_ receptor-dependent release from the endoplasmic reticulum and by plasma membrane channels, emerges as the key integrative signal through which astrocytes sense and respond to synaptic activity and, in turn, modulate plasticity. The review also addresses the structural dynamics of PAPs themselves: astrocytic processes undergo activity-dependent morphological remodeling, expanding toward or retracting from active synapses in ways that bidirectionally regulate synaptic efficacy and the induction thresholds for LTP and LTD. This structural plasticity of astrocytic processes is now understood to be regulated by cytoskeletal proteins, adhesion molecules, and signaling cascades, including Rho GTPases and EphA/ephrin pathways that respond to local synaptic activity and neuromodulatory signals. Beyond individual synapses, the review discusses how astrocytic networks, coupled through gap junctions formed by connexins 30 and 43, coordinate plasticity across broader spatial scales, integrating signals from multiple synaptic inputs and translating them into coherent modulatory responses at the circuit level. The authors further highlight how astrocytic dysfunction, including impaired glutamate clearance, disrupted calcium signaling, and altered gliotransmitter release, contributes to synaptic pathology in aging, AD, and psychiatric disorders, positioning astrocytic failure as a potentially primary rather than merely secondary driver of cognitive impairment. The review by Yamamoto and Takano thus reinforces the tripartite synapse as a fundamental organizational unit of information storage and retrieval in the brain, and makes a compelling case for astrocyte-targeted interventions as a largely unexplored therapeutic avenue for disorders of learning, memory, and emotional regulation [5].

## 6. Conclusions and Future Directions

The contributions assembled in this Special Issue collectively advance our understanding of synaptic plasticity across multiple scales of analysis, from the post-transcriptional regulation of individual synaptic proteins to the system-level consequences of neuroinflammation and metabolic disease. Reading these works together reveals several cross-cutting themes that speak to the current state of the field and its most promising future directions.

The boundaries between synaptic plasticity and neuropsychiatric phenomenology are increasingly permeable. The findings of Panuccio et al. [1] emphasize that motivational deficits, apathy, and reward-circuit dysfunction are not merely behavioral epiphenomena of synaptic pathology but are mechanistically linked to it, emerging early in the disease course and potentially serving as sensitive biomarkers of circuit dysfunction preceding overt cognitive decline. This reframing of AD as a disorder of affective disconnection, alongside the classical narrative of memory failure, may have important implications for early diagnosis and design of therapeutic interventions targeting non-cognitive symptoms.

Furthermore, the molecular diversity of synapses is regulated by signaling systems not previously appreciated for this role. The discovery by Innocenzi et al. [3] that CB1 receptor signaling dynamically shapes neurexin splice variant composition via the SLM2-dependent splicing machinery reveals a previously unsuspected layer of synaptic regulation, situated at the intersection of neuromodulation and post-transcriptional gene control. These findings invite a broader reconsideration of how the chemical milieu of active neural circuits shaped by endocannabinoids, neuromodulators, and experience continuously sculpts the molecular identity of synapses in ways that feed back onto circuit function.

The convergence of transcriptomic findings across distinct genetic backgrounds in the PKU models studied by Fiori et al. [2] underscores a general principle of considerable translational value: robust, disease-relevant molecular signatures can be identified when animal models are compared systematically rather than studied in isolation. The shared PFC transcriptional dysregulation centered on translational stress and impaired myelination, validated behaviorally by a novel working memory paradigm, provides a molecular entry point for the development of targeted interventions in PKU and potentially in other metabolic disorders affecting prefrontal function.

More broadly, the contributions of Imbriani et al. [4] and Yamamoto and Takano [5] make a compelling case that the synapse is not a neuronal structure that happens to be regulated by glia and immune cells, but rather a fundamentally tripartite computational unit whose properties emerge from the coordinated activity of neurons, astrocytes, microglia, and the wider neuroimmune milieu. Astrocytic process dynamics, gliotransmitter release, cytokine-mediated synaptic scaling, and complement-dependent pruning are not peripheral modulatory influences but core mechanisms of synaptic plasticity, whose dysregulation is sufficient to drive cognitive and behavioral pathology across a wide spectrum of conditions.

Looking forward, several research priorities emerge from the collection of the present Special Issue. The development of circuit-specific and temporally targeted interventions, whether lipid-based neuromodulators such as PEA, splicing-directed molecular tools, or non-pharmacological multimodal stimulation strategies, represents a promising direction that moves beyond widespread approaches toward precision modulation of defined synaptic substrates. The identification of molecular targets shared across disease models and genetic backgrounds, as illustrated both by the PKU transcriptomics and by the convergent neuroimmune mechanisms reviewed by Imbriani et al. [4], offers a rational basis for the design of broadly applicable yet mechanistically grounded treatments. Moreover, clarifying how neuromodulatory signals, activity patterns, and glial states interact to regulate the post-transcriptional diversity of synaptic proteins, as exemplified by the CB1-neurexin axis, will allow us to understand how experience shapes synaptic identity and how its disruption may contribute to disease.

We thank all authors for their outstanding contributions to this Special Issue, and the expert reviewers for their rigorous and constructive engagement with each manuscript. We are confident that this collection will serve as a valuable point of reference for researchers working at the intersection of synaptic biology, neuroscience, and translational medicine, and that it will stimulate further investigation into the synaptic foundations of learning, memory, and their disorders.

## Abbreviations

The following abbreviations are used in this manuscript:

AD	Alzheimer’s disease
APP	Amyloid precursor protein
BDNF	Brain-derived neurotrophic factor
CB1	Cannabinoid receptor 1
CPP	Conditioned place preference
EC	Entorhinal cortex
HPA	Hyperphenylalaninemia
LTD	Long-term depression
LTP	Long-term potentiation
PAH	Phenylalanine hydroxylase
PAPs	Perisynaptic astrocytic processes
PEA	Palmitoylethanolamide
PKU	Phenylketonuria
PPARα	Peroxisome proliferator-activated receptor alpha
PFC	Prefrontal cortex
